# Curcumin alleviates renal fibrosis in chronic kidney disease by targeting the circ_0008925-related pathway

**DOI:** 10.1080/0886022X.2024.2444393

**Published:** 2025-03-04

**Authors:** Peng An, Xingyao Li, Yanhong Zhao, Liuyun Li, Yafeng Wang, Wenfang Wang, Tao Zhang, Sicen Wang, Xili Wu

**Affiliations:** a Department of Traditional Chinese Medicine, the Second Affiliated Hospital of Xi’an Jiaotong University, Xi’an, Shaanxi, China; b School of Pharmacy, Xi’an Jiaotong University, Xi’an, Shaanxi, China

**Keywords:** Renal fibrosis, curcumin, circ_0008925, miR-204-5p, IL6ST

## Abstract

**Background:**

Curcumin has been shown to inhibit renal fibrosis, but whether curcumin mediates renal fibrosis progression by regulating the circular RNA (circRNA)-related pathway remain unclear.

**Methods:**

TGF-β1 was used to construct renal injury and fibrosis cell model. Cell growth was evaluated by cell counting kit 8 assay, EdU assay and flow cytometry. Fibrosis marker and interleukin 6 signal transducer (IL6ST) protein levels were measured using western bolt analysis. Inflammation factor concentrations were determined by ELISA. Circ_0008925, miR-204-5p and IL6ST expression was assessed by qRT-PCR. Unilateral ureteral obstruction (UUO) mice models were constructed to assess the role of curcumin *in vivo*.

**Results:**

Curcumin treatment alleviated TGF-β1-induced HK-2 cell apoptosis, inflammation and fibrosis *in vitro*, as well as relieved renal injury in UUO mice models *in vivo*. Circ_0008925 was highly expressed in TGF-β1-induced HK-2 cells and its expression was inhibited by curcumin. Circ_0008925 could sponge miR-204-5p to positively regulate IL6ST. The inhibition effect of curcumin on TGF-β1-induced HK-2 cell injury and fibrosis was reversed by circ_0008925 overexpression, miR-204-5p inhibitor or IL6ST upregulation. Besides, circ_0008925 knockdown inhibited TGF-β1-induced HK-2 cell injury and fibrosis by suppressing IL6ST expression.

**Conclusion:**

Curcumin relieved renal fibrosis through regulating circ_0008925/miR-204-5p/IL6ST axis.

## HIGHLIGHTS

1. Curcumin inhibits renal fibrosis by reducing circ_0008925 expression.
2. Circ_0008925 sponges miR-204-5p.
3. MiR-204-5p targets IL6ST.
4. Curcumin regulates the circ_0008925/miR-204-5p/IL6ST axis.

## Introduction

Chronic kidney disease (CKD), refers to various degrees of chronic renal structural and functional disorders, has become one of the main diseases threatening public health [1,2]. Renal fibrosis can lead to renal function loss and failure, characterized by glomerular mesangial cell proliferation, renal tubule atrophy, inflammatory cell infiltration, and excessive accumulation of extracellular matrix [3–5]. There is no effective treatment for renal fibrosis now, which can only be delayed by protecting the kidney [6]. Therefore, clarifying the underlying molecular mechanism may provide a theoretical basis for treating renal fibrosis.

Curcumin is a plant polyphenol extracted from turmeric, and is also the most important active component of turmeric [7,8]. Curcumin has anti-inflammatory, antioxidant, anti-tumor, and other pharmacological effects [9,10]. Curcumin has also been found to have kidney protective and anti-fibrosis properties [11,12]. However, the potential molecular mechanism of curcumin inhibiting renal fibrosis remains to be further studied.

Circular RNAs (circRNAs) has been confirmed to regulate human disease progression [13,14]. CircRNA acts as a microRNA (miRNA) sponge to mediate gene expression [15,16]. Many studies have suggested that circRNA is involved in the progression of renal fibrosis [17,18]. Circ_0008925 is located at chr6: 108222573-108246136, and is derived by the SEC63 gene. Circ_0008925 was remarkably overexpressed in urinary exosomes of patients with renal fibrosis and could be used as a potential biomarker of renal fibrosis [19]. However, the role and mechanism of circ_0008925 in renal fibrosis remain unclear.

In this, curcumin had an inhibition on circ_0008925 expression and could alleviate TGF-β1-induced HK-2 injury and fibrosis. Hence, we proposed hypothesis that curcumin hindered circ_0008925 expression to alleviate renal fibrosis. In addition, we also explored the circ_0008925-mediated miRNA/mRNA regulatory axis and improved the potential molecular mechanism of curcumin regulated renal fibrosis.

## Materials and methods

### Cell culture and treatment

HK-2 cells (ATCC, USA) were maintained in DMEM plus 10% FBS and 1% penicillin/streptomycin (Invitrogen, USA). Cells were stimulated with 5 ng/mL TGF-β1 for 48 h to induce renal cell injury and fibrosis model as previously described [20]. In addition, cells were treated with TGF-β1 and different concentrations of curcumin to screen the optical concentration of curcumin.

### Cell counting kit 8 (CCK8) assay

HK-2 cells seeded into 96-well plates were cultured for 48 h. After incubated with CCK8 reagent (Beyotime, China), cell viability was assessed *via* a microplate reader at 450 nm.

### EdU assay

As previously described [21], HK-2 cells in 6-well plates were stained by EdU solution and DAPI solution (RiboBio, China). Fluorescent images were captured under a microscope to count EdU positive cell rate.

### Flow cytometry

As previously described [22], HK-2 cells suspended with Binding buffer were stained with Annexin V-FITC and PI solution (BD Biosciences, USA). Cell apoptosis was analyzed by flow cytometry with FlowJo software (BD Biosciences, USA).

### Western blot (WB) analysis

As previously described [23], total proteins were extracted by RIPA buffer (Beyotime, China), separated using SDS-PAGE gels followed by transferred to PVDF membranes. After incubated with anti-α-SMA (ab5694, 1:1000, Abcam, USA), anti-collagen I (ab34710, 1:1000, Abcam, USA), anti-interleukin 6 signal transducer (IL6ST) (ab283685, 1:1000, Abcam, USA) and anti-GAPDH (ab9485, 1:2500, Abcam, USA), membrane was incubated with secondary antibody. Protein bands were visualized using ECL reagents (Beyotime, China).

### ELISA assay

After centrifuged, cell supernatant was collected. The concentrations of IL-6 and TNF-α in cell supernatant were determined by Human IL-6 and TNF-α ELISA kits (Solarbio, China) according to kit instructions.

### qRT-PCR

As previously described [24], total RNAs were extracted using TRIzol reagent (Invitrogen, USA), and cDNA was synthesized using a cDNA Synthesis Kit (Beyotime, China). Then, SYBR Green (Takara, China) was mixed with cDNA and specific primers (Table 1) to perform PCR. Fold change was analyzed by the 2^−ΔΔCT^ method.

**Table 1.** Primer sequences used for qRT-PCR.

Name		Primers for qRT-PCR (5′–3′)
hsa_circ_0008925	Forward	GTTATGGCTGTCCTTGGGAGT
	Reverse	AGCAGAACTATTTTCCCTGTGA
IL6ST	Forward	TGGCTAGTGCAAGCCTTGTT
	Reverse	AGGTGACACTGGATGCTGTT
miR-204-5p	Forward	GTATGAGTTCCCTTTGTCATCCTA
	Reverse	CTCAACTGGTGTCGTGGAG
GAPDH	Forward	GACCACAGTCCATGCCATCAC
	Reverse	ACGCCTGCTTCACCACCTT
U6	Forward	GCTTCGGCAGCACATATACTAA
	Reverse	AACGCTTCACGAATTTGCGT

### Identification of circ_0008925

RNA extracted from HK-2 cells was additionally co-incubated with RNase R and used for qRT-PCR. To explore whether circ_0008925 had a poly-A tail, cDNA was synthesized by random primers or oligo (dT)_18_ primers in reverse transcription and then used for qRT-PCR. The cytoplasm and nuclear RNAs from HK-2 cells were isolated by PARIS Kit (Invitrogen, USA), and qRT-PCR was performed to detect circ_0008925 expression in the cytoplasm and nuclear.

### Cell transfection

Circ_0056686 pCD5 overexpression vector or small interfering RNA (si-circ_0008925), miR-204-5p mimic, inhibitor (anti-miR-204-5p), pcDNA IL6ST overexpression vector and their negative controls (RiboBio, China) were transfected into HK-2 cells using lipofectamine 3000 (Invitrogen, USA).

### Dual-luciferase reporter assay

As previously described [24], the wild-type and mutant-type segments of circ_0008925 or IL6ST 3’UTR were inserted into the pGL3 reporter vector. HK-2 cells were co-transfected with WT/MUT-circ_0008925 vectors or WT/MUT-IL6ST 3’UTR vectors with miR-204-5p mimic/miR-NC. Relative luciferase activity was quantified using a Dual-Lucy Assay Kit (Solarbio, China) 48 h later.

### RNA pull-down assay

As previously described [24], the bio-miR-204-5p probe and bio-miR-NC probe (RiboBio, China) were transfected into HK2 cells for 48 h. Cell lysates were collected and incubated with Dynabeads M-280 Streptavidin (Invitrogen, USA). Then, the enrichments of bound circ_0008925 and IL6ST in each probe were detected by qRT-PCR.

### Unilateral ureteral obstruction (UUO) models

As previously described [25], male C57BL/6 mice (Vital River, China) were anesthetized with 1% pentobarbital and ligated the left ureter with 4-0 silk thread to construct UUO model (*n* = 5). Sham-operated mice (*n* = 5) underwent the same procedure without ligation. Mice in the UUO+curcumin group (*n* = 5) were administered curcumin by gastric gavage at a daily dose of 50 mg/kg. After 14 d, blood samples and kidney tissues were collected after mice were sacrificed. The kidney samples were paraffin embedded and cut to 5 mm thick slices for hematoxylin and eosin (HE) and MASSON staining. The concentrations of IL-6 and TNF-α in mice blood samples were detected by corresponding Mouse ELISA Kits (Solarbio, China). Animal studies were approved by the Animal Ethics Committee of the Second Affiliated Hospital of Xi’an Jiaotong University.

### Statistical analysis

Data are shown as means ± SD. Comparisons between groups were analyzed by Student’s *t*-tests or analysis of variance using GraphPad 7.0 software. *p* < .05 was regarded as statistically significant.

## Results

### Curcumin inhibited TGF-β1-stimulated HK2 cell injury and fibrosis

**Figure 1.**
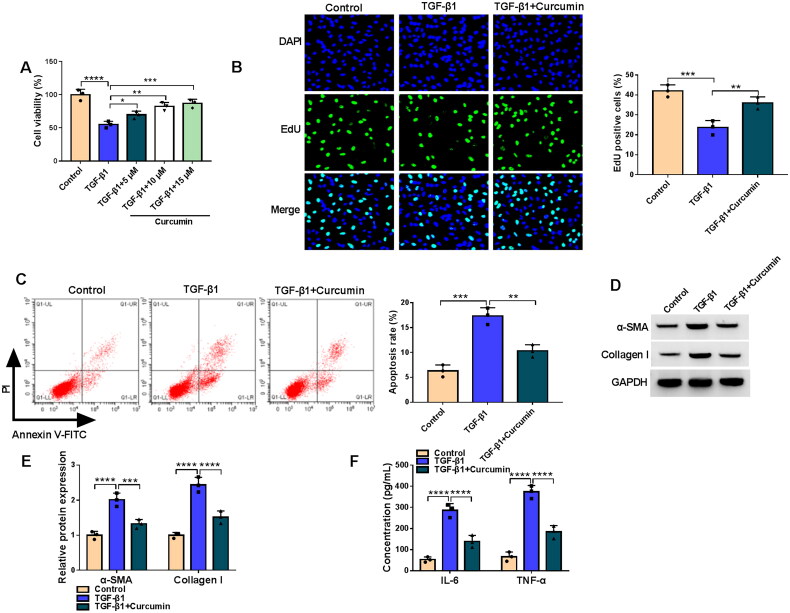
Effects of curcumin on TGF-β1-stimulated HK2 cell injury and fibrosis. (A) CCK8 assay was used to detect cell viability in HK2 cells treated with TGF-β1 and different concentration of curcumin. (B-F) HK2 cells were treated with 10 μM curcumin and then induced by TGF-β1. (B) EdU assay was used to measure EdU positive cell rate. (C) Apoptosis rate was detected by flow cytometry. (D-E) WB analysis was performed to analyze protein expression. (F) The concentrations of IL-6 and TNF-α were examined by ELISA assay. *p < .05, **p < .01, ***p < .001, ****p < .0001.

Firstly, we assessed the effect of curcumin on TGF-β1-stimulated HK2 cell injury and fibrosis. Under TGF-β1 treatment, HK2 cell viability was markedly reduced. After treated with different concentrations of curcumin, we found a gradual increase in cell viability (Figure 1A). In the follow-up study, 10 μM curcumin was used for cell treatment. TGF-β1 treatment inhibited EdU positive cell rate, and this effect was abolished by curcumin (Figure 1B). In addition, TGF-β1 promoted HK2 cell apoptosis rate, fibrosis markers (α-SMA and Collagen I) protein expression, and inflammatory factors (IL-6 and TNF-α) concentrations. However, curcumin treatment also alleviated TGF-β1-induced apoptosis, fibrosis and inflammation in HK2 cells (Figure 1C–F). Thus, we believed that curcumin might alleviate renal cell injury and fibrosis.

### Circ_0008925 was highly expressed in TGF-β1-stimulated HK2 cells and its expression was regulated by curcumin

**Figure 2.**
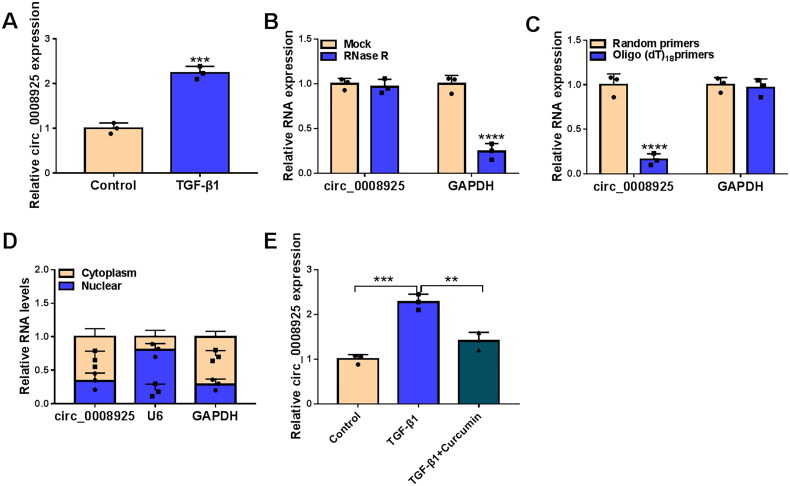
Circ_0008925 expression in TGF-β1-stimulated HK2 cells and curcumin treatment. (A) Circ_0008925 expression was detected by qRT-PCR in HK2 cells treated with or without TGF-β1. (B) After RNA was treated with RNase R, qRT-PCR was used to detect circ_0008925 and GAPDH expression. (C) After amplified by random primers and oligo (dT)_18_ primers, circ_0008925 and GAPDH expression was examined by qRT-PCR. (D) Subcellular localization analysis was used to analyze the distribution of circ_0008925 in the cytoplasm and nuclear of HK2 cells. (E) Circ_0008925 expression was determined by qRT-PCR in HK2 treated with TGF-β1 and curcumin. **p < .01, ***p < .001, ****p < .0001.

Compared to non-treated cells, circ_0008925 was upregulated in TGF-β1-induced HK2 cells (Figure 2A). To confirm the circular feature of circ_0008925, we measured circ_0008925 expression under RNase R treatment and oligo (dT)_18_ primers treatment. The results showed that circ_0008925 expression had not any changed after RNase R treatment and it cannot be amplified by oligo (dT)_18_ primers (Figure 2B–C), suggesting that circ_0008925 could resist to RNase R digestion and did not contain poly-A tail. Besides, subcellular localization analysis and cellular immunofluorescence staining showed that circ_0008925 was mainly distributed in the cytoplasm (Figure 2D and Supplementary Figure 1). Curcumin treatment reduced circ_0008925 expression in TGF-β1-induced HK2 cells (Figure 2E), showing that curcumin might regulate renal fibrosis through circ_0008925.

### Circ_0008925 overexpression reversed the inhibition of curcumin on TGF-β1-mediated HK2 cell injury and fibrosis

**Figure 3.**
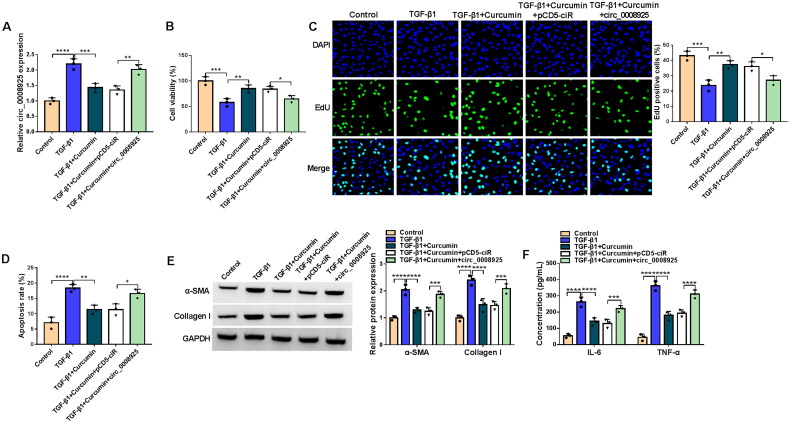
Effects of curcumin and circ_0008925 overexpression on TGF-β1-mediated HK2 cell injury and fibrosis. HK2 cells were transfected with pCD5-ciR or pCD5 circ_0008925 overexpression vector, and then treated with curcumin and TGF-β1. (A) Circ_0008925 expression was assessed by qRT-PCR. CCK8 assay (B), EdU assay (C) and flow cytometry (D) were used to determine cell proliferation and apoptosis. (E) Protein expression was measured by WB analysis. (F) ELISA assay was employed to examine the concentrations of IL-6 and TNF-α. *p < .05, **p < .01, ***p < .001, ****p < .0001.

To confirm whether curcumin regulated renal injury and fibrosis by mediating circ_0008925 expression, HK2 cells were transfected with pCD5 circ_0008925 overexpression vector and then treated with curcumin and TGF-β1. The addition of pCD5 circ_0008925 overexpression vector increased circ_0008925 expression inhibited by curcumin in TGF-β1-induced HK2 cells (Figure 3A). Overexpressed circ_0008925 reversed the increasing effect of curcumin on viability and EdU positive cell rate in TGF-β1-induced HK2 cells (Figure 3B,C). Also, the upregulation of circ_0008925 eliminated the suppressive effect of curcumin on the apoptosis rate, fibrosis markers protein expression, and inflammatory factors concentrations in TGF-β1-induced HK2 cells (Figure 3D–F). The above data revealed that curcumin might suppress renal injury and fibrosis *via* decreasing circ_0008925 expression.

### Circ_0008925 served as miR-204-5p sponge

**Figure 4.**
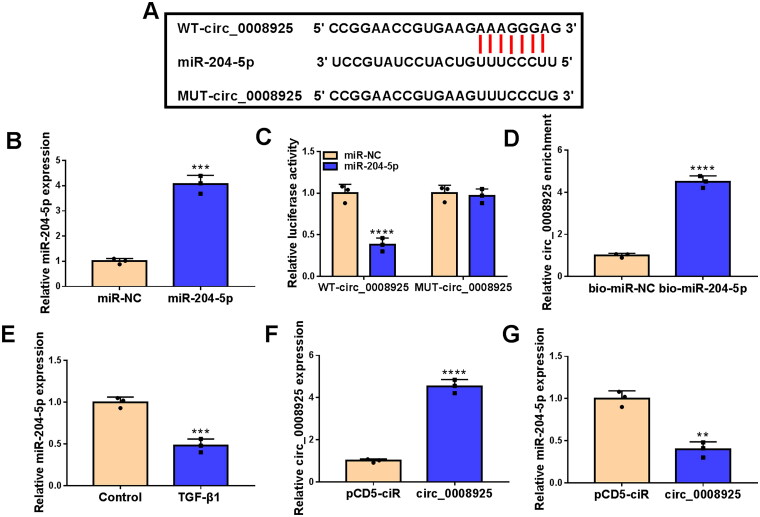
Circ_0008925 sponged miR-204-5p. (A) The sequences of WT/MUT-circ_0008925 vectors were shown. (B) The transfection efficiency of miR-204-5p mimic was confirmed by qRT-PCR. Dual-luciferase reporter assay (C) and RNA pull-down assay (D) were used to confirm the interaction between circ_0008925 and miR-204-5p. (E) MiR-204-5p expression was analyzed by qRT-PCR in HK2 cells treated with or without TGF-β1. (F) The transfection efficiency of pCD5 circ_0008925 overexpression vector was confirmed by qRT-PCR. (G) MiR-204-5p expression was examined by qRT-PCR in HK2 cells transfected with pCD5-ciR or pCD5 circ_0008925 overexpression vector. **p < .01, ***p < .001, ****p < .0001.

Using starBase2.0 software, circ_0008925 had binding sites with miR-204-5p (Figure 4A). MiR-204-5p mimic was used to overexpress miR-204-5p (Figure 4B). Only the luciferase activity of the WT-circ_0008925 vector was reduced by the miR-204-5p mimic (Figure 4C), and the highly enrichment of circ_0008925 was observed in the bio-miR-204-5p probe (Figure 4D). These data confirmed the interaction between circ_0008925 and miR-204-5p. The miR-204-5p level was decreased in TGF-β1-induced HK2 cells (Figure 4E). After confirmed that the pCD5 circ-0008925 overexpression vector could enhance circ_0008925 expression in HK2 cells (Figure 4F), we measured miR-204-5p expression, and found that circ_0008925 upregulation suppressed miR-204-5p expression (Figure 4G). Therefore, we confirmed that circ_0008925 sponged miR-204-5p to negatively regulate its expression.

### Curcumin suppressed TGF-β1-induced HK2 cell injury and fibrosis by increasing miR-204-5p

**Figure 5.**
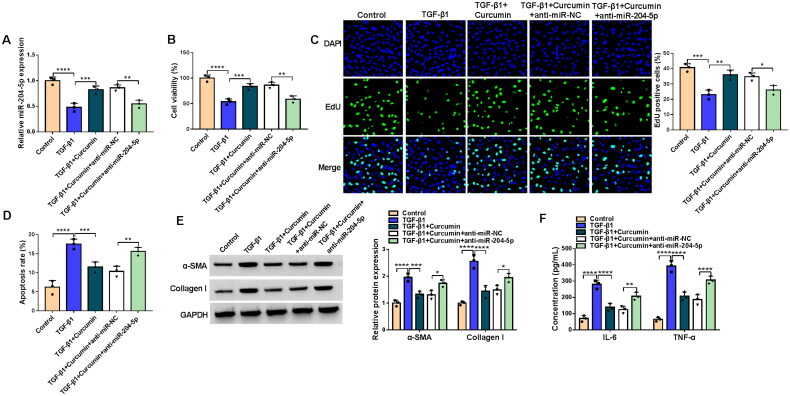
Effects of curcumin and anti-miR-204-5p on TGF-β1-induced HK2 cell injury and fibrosis. HK2 cells were transfected with anti-miR-NC or anti-miR-204-5p, and then treated with curcumin and TGF-β1. (A) MiR-204-5p expression was tested by qRT-PCR. Cell proliferation and apoptosis were detected by CCK8 assay (B), EdU assay (C) and flow cytometry (D). (E) WB analysis was performed to detect protein expression. (F) The concentrations of IL-6 and TNF-α were tested using ELISA assay. *p < .05, **p < .01, ***p < .001, ****p < .0001.

Curcumin treatment enhanced miR-204-5p level in TGF-β1-induced HK2 cells (Figure 5A). To explore whether curcumin regulated TGF-β1-induced HK2 cell injury by mediating miR-204-5p expression, HK2 cells were transfected with anti-miR-204-5p and treated with curcumin and TGF-β1. The transfection of anti-miR-204-5p reduced miR-204-5p expression under curcumin treatment in TGF-β1-induced HK2 cells (Figure 5A). As presented in Figure 5B,C, miR-204-5p inhibitor eliminated the enhancing effect of curcumin on the viability and EdU positive cell rate of TGF-β1-induced HK2 cells. Te inhibition of curcumin on the apoptosis rate, fibrosis markers protein expression and inflammatory factors concentrations in TGF-β1-induced HK2 cells also were reversed by a miR-204-5p inhibitor (Figure 5D–F). Above all, we confirmed that curcumin relieved renal fibrosis through increasing miR-204-5p expression.

### MiR-204-5p targeted IL6ST

**Figure 6.**
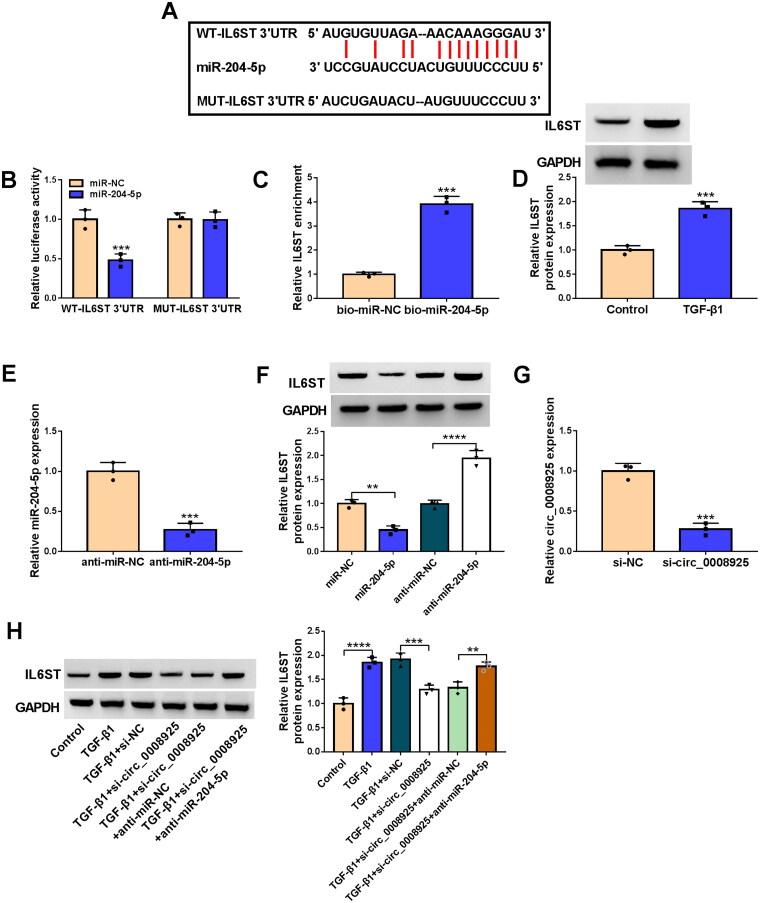
MiR-204-5p targeted IL6ST. (A) The sequences of WT/MUT-IL6ST 3’UTR vectors were shown. Dual-luciferase reporter assay (B) and RNA pull-down assay (C) were used to confirm the interaction between miR-204-5p and IL6ST. (D) IL6ST protein expression was analyzed by WB analysis in HK2 cells treated with or without TGF-β1. (E) The transfection efficiency of anti-miR-204-5p was confirmed by qRT-PCR. (F) IL6ST protein expression was examined by WB analysis in HK2 cells transfected with miR-204-5p mimic or inhibitor. (G) QRT-PCR was used to confirm the transfection efficiency of si-circ_0008925. (H) IL6ST protein expression was tested by WB analysis in TGF-β1-induced HK2 cells co-transfected with si-circ_0008925 and anti-miR-204-5p. **p < .01, ***p < .001, ****p < .0001.

MiR-204-5p had binding sites with IL6ST 3’UTR predicted by starBase v2.0 software (Figure 6A). MiR-204-5p mimic inhibited the luciferase activity of WT-IL6ST 3’UTR vector (Figure 6B), and IL6ST enrichment was significantly increased in the bio-miR-204-5p probe (Figure 6C), confirming the interaction between miR-204-5p and IL6ST. TGF-β1 treatment enhanced IL6ST expression in HK2 cells (Figure 6D). The anti-miR-204-5p was used to decrease miR-204-5p expression in HK2 cells (Figure 6E). Further analysis confirmed that IL6ST protein expression was reduced by miR-204-5p mimic and increased by its inhibitor (Figure 6F). Then, si-circ_0008925 was constructed to reduce circ_0008925 expression in HK2 cells (Figure 6G). Function analysis showed that circ_0008925 knockdown inhibited IL6ST protein expression in TGF-β1-induced HK2 cells, and this effect was reversed by miR-204-5p inhibitor (Figure 6H). Above all, we confirmed that circ_0008925 positively regulated IL6ST expression by sponging miR-204-5p.

### Circ_0008925 knockdown inhibited TGF-β1-induced HK2 cell injury and fibrosis by reducing IL6ST expression

**Figure 7.**
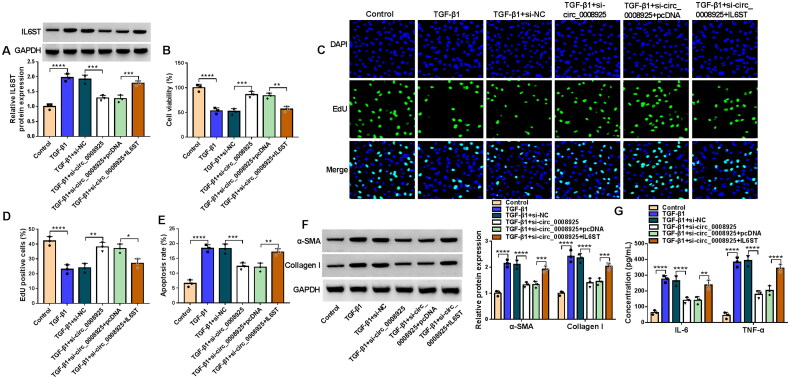
Effects of circ_0008925 knockdown and IL6ST overexpression on TGF-β1-induced HK2 cell injury and fibrosis. HK2 cells were co-transfected with si-circ_0008925 and pcDNA IL6ST overexpression vector followed by treated with TGF-β1. (A) IL6ST protein expression was measured by WB analysis. CCK8 assay (B), EdU assay (C-D) and flow cytometry (E) were performed to measure cell proliferation and apoptosis. (F) Protein expression was tested using WB analysis. (G) ELISA assay was used to test the concentrations of IL-6 and TNF-α. *p < .05, **p < .01, ***p < .001, ****p < .0001.

To explore whether circ_0008925 regulated renal injury and fibrosis by regulating IL6ST, we performed rescue experiments. The addition of pcDNA IL6ST overexpression vector enhanced IL6ST expression reduced by si-circ_0008925 in TGF-β1-induced HK2 cells (Figure 7A). Function experiments indicated that circ_0008925 knockdown promoted the viability and EdU positive cell rate in TGF-β1-induced HK2 cells, while IL6ST overexpression reversed this effect (Figure 7B–D). Also, downregulated circ_0008925 could inhibit the apoptosis rate, the fibrosis markers protein expression and the concentrations of inflammatory factors in TGF-β1-induced HK2 cells, while these effects also were overturned by overexpressing IL6ST (Figure 7E–G). Therefore, circ_0008925 might promote renal injury and fibrosis by increasing IL6ST expression.

### Curcumin regulated TGF-β1-mediated HK2 cell injury and fibrosis via repressing IL6ST expression

**Figure 8.**
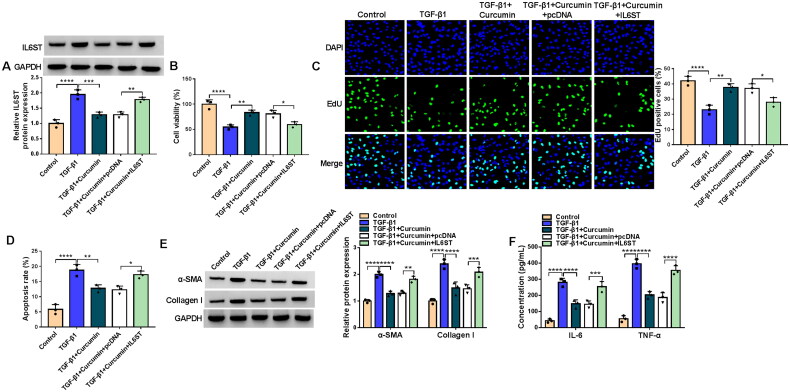
Effects of curcumin and IL6ST on TGF-β1-mediated HK2 cell injury and fibrosis. HK2 cells were transfected with pcDNA or pcDNA IL6ST overexpression vector, and then treated with curcumin and TGF-β1. (A) WB analysis was used to detect IL6ST protein expression. Cell proliferation and apoptosis were determined using CCK8 assay (B), EdU assay (C) and flow cytometry (D). (E) Protein expression was measured using WB analysis. (F) The concentrations of IL-6 and TNF-α were determined by ELISA assay. *p < .05, **p < .01, ***p < .001, ****p < .0001.

To further confirm our conclusion, we explored whether IL6ST participated in the regulation of curcumin on TGF-β1-induced HK2 cell injury and fibrosis. Curcumin markedly decreased IL6ST protein expression, and the pcDNA IL6ST overexpression vector abolished this effect (Figure 8A). IL6ST overexpression eliminated the enhancing effect of curcumin on proliferation, as well as the reducing effect on apoptosis, fibrosis markers protein expression and the concentrations of inflammatory factors (Figure 8B–F). Hence, we confirmed that curcumin alleviated renal cell injury and fibrosis *via* reducing IL6ST expression.

### Curcumin alleviated renal fibrosis in UUO-induced mice via regulating circ_0008925/miR-204-5p/IL6ST axis

**Figure 9.**
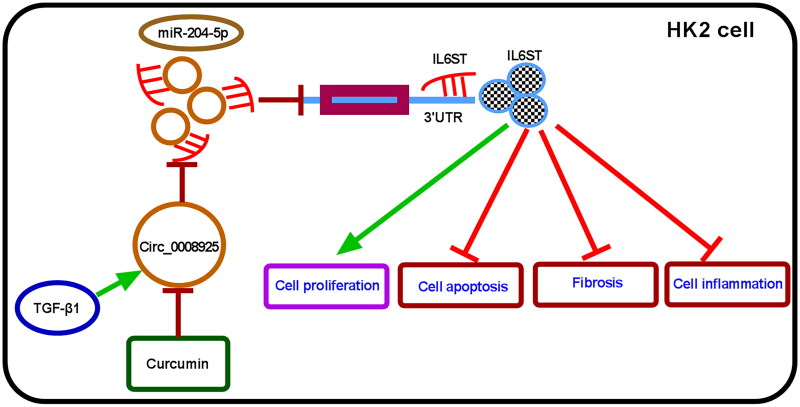
The mechanism diagram of this study.

For further confirming the anti-fibrosis roles of curcumin, we performed animal study. Through HE and MASSON staining, we found that UUO induction could promote renal tubular dilation and fibrotic deposition, while curcumin treatment improved renal tubular dilation and reduced fibrotic deposition in UUO-induced mice (Supplementary Figure 2A). In UUO-induced mice, α-SMA, Collagen I, IL-6 and TNF-α levels were significantly enhanced in kidney tissues, while curcumin treatment reversed these effects (Supplementary Figure 2B,C). In addition, we observed the high circ_0008925, IL6ST, and low miR-204-5p expression in kidney tissues of UUO-induced mice, while these effects were eliminated by curcumin (Supplementary Figure 2D–F). Above all, we pointed out that curcumin might inhibit renal fibrosis through the circ_0008925/miR-204-5p/IL6ST axis (Figure 9).

## Discussion

Many study has been reported the anti-fibrosis effect of curcumin. Previous research showed that curcumin could alleviate renal fibrosis by restraining inflammatory response [25]. Besides, curcumin hindered the EMT process to relieve the renal fibrosis process [26]. Here, we explored the role of curcumin in renal fibrosis. In TGF-β1-induced HK-2 cell apoptosis, inflammation and fibrosis, we confirmed that curcumin treatment suppressed cell injury and fibrosis. In animal study, we also confirmed the anti-fibrosis effect of curcumin in renal.

Although the anti-fibrosis effect of curcumin has been confirmed, but the specific molecular regulatory mechanisms of curcumin in renal fibrosis remains to be revealed. As an important regulatory factor, circRNA is a potential marker for many diseases, such as cardiovascular diseases [27], inflammatory diseases [28] and neurological diseases [29]. However, it has not been reported whether curcumin regulates renal fibrosis by regulating circRNA-related pathways. Here, we found that curcumin decreased the expression of circ_0008925, which was an overexpressed circRNA in patients with renal fibrosis [19]. After overexpressing circ_0008925, the suppressive effect of curcumin on TGF-β1-induced HK-2 cell injury and fibrosis were partially eliminated, confirming that curcumin relieved renal fibrosis by decreasing circ_0008925. Function analysis suggested that circ_0008925 knockdown also abolished TGF-β1-induced HK-2 cell injury and fibrosis, which indicated that circ_0008925 indeed contributed to the renal fibrosis process. In short, this study demonstrated for the first time that curcumin mediates renal fibrosis by regulating circ_0008925, which provided a new route for subsequent studies on the intrinsic mechanisms of renal fibrosis.

Through bioinformatics analysis, circ_0008925 had been confirmed to be a ceRNA for miR-204-5p. In many types of cancer, miR-204-5p acts as a tumor suppressor to regulate cancer development [30,31]. Upregulation of miR-204-5p alleviated inflammatory response in cerebral ischemia and reperfusion neuron injury [32]. MiR-204-5p knockdown is thought to aggravate kidney injury, so it is considered to be an important miRNA to protect the kidney from chronic injury [33]. Recently study suggested that miR-204 inhibited oxidative stress in HK-2 cells induced by H_2_O_2_ [34]. In acute kidney injury, miR-204-5p also had an inhibition on hypoxic-induced HK-2 cell apoptosis and inflammation [35]. Here, miR-204-5p was sponged by circ_0008925, and its level could be promoted by curcumin. In the rescue experiments, miR-204-5p knockdown revoked the negatively regulation of curcumin on TGF-β1-induced HK-2 cell injury and fibrosis. Therefore, we believed that curcumin inhibited renal fibrosis by mediating the circ_0008925/miR-204-5p axis.

With the rapid development of sequencing technology and bioinformatics methods, researchers have discovered different classes of non-coding RNAs (ncRNAs) [36]. Although ncRNAs do not encode proteins, they indirectly regulate a variety of biological processes, including cell metabolism and development [37]. It has been shown that circRNAs can sponge miRNAs to regulate the mRNAs expression, thereby modulating the cell physiological state [38]. To improve the assumption of the circRNA/miRNA/mRNA axis, we explored the target of miR-204-5p and confirmed that IL6ST could interact with miR-204-5p. IL6ST, also known as gp130, is a cytokine like receptor that is a founding member of a transmembrane protein [39]. Studies had shown that the Il6ST-related signaling axis mediated cancer progression, and targeting IL6ST might be a potential approach for cancer therapy [40]. In previous studies, IL6ST had been shown to be involved in fibrosis formation in multiple organs, including pulmonary [41] and liver [42]. IL6ST overexpression promoted fibrosis marker expression in high glucose-induced HK-2 cells [24]. Here, circ_0008925 sponged miR-204-5p to positively regulate IL6ST. IL6ST upregulation abolished si-circ_0008925-mediated the inhibition on TGF-β1-induced HK-2 cell injury and fibrosis, verifying that circ_0008925 promoted renal fibrosis by positively regulating IL6ST level. In addition, IL6ST expression was decreased by curcumin, and its overexpression also overturned the function of curcumin on renal fibrosis, confirming the conclusion of curcumin regulated circ_0008925/miR-204-5p/IL6ST axis in renal fibrosis.

This study reveals for the first time that curcumin mediates renal fibrosis by regulating circ_0008925. In addition, the circ_0008925/miR-204-5p/IL6ST axis is also proposed by us for the first time, which is a major discovery of this study. However, there are some limitations to this study. We have not yet revealed the downstream pathway of the circ_0008925/miR-204-5p/IL6ST axis to further refine our experimental results. In future studies, we will explore the downstream pathway of the circ_0008925/miR-204-5p/IL6ST axis to provide more evidence for an in-depth understanding of the anti-fibrotic effects of curcumin.

In summary, our study revealed the underlying molecular mechanism by which curcumin inhibited renal fibrosis. We showed that curcumin suppressed TGF-β1-induced HK-2 cell injury and fibrosis by circ_0008925/miR-204-5p/IL6ST axis. Our findings not only provide new evidence for curcumin to be used in anti-fibrosis therapy, but also the proposed circ_0008925-related regulatory axis provides a potential molecular target for treating renal fibrosis.

## Supplementary Material

Supplementary Figure 1.tif

Supplementary Figure 2 new.tif

Figure 8 revised.tif

Figure 3 revised.tif

Figure 6 revised.tif

Figure 2 revised.tif

Figure 4 revised.tif

Figure 1 revised.tif

Figure 7 revised.tif

Figure 5 revised.tif

Figure 9.tif

All original drawing of WB.pdf
