# Supplementary figures and images for: Curcumin alleviates renal fibrosis in chronic kidney disease by targeting the circ_0008925-related pathway
Source: Ren Fail. 2025 Mar 4;47(1):2444393. doi: 10.1080/0886022X.2024.2444393 (PMC11884099; doi:10.1080/0886022X.2024.2444393)

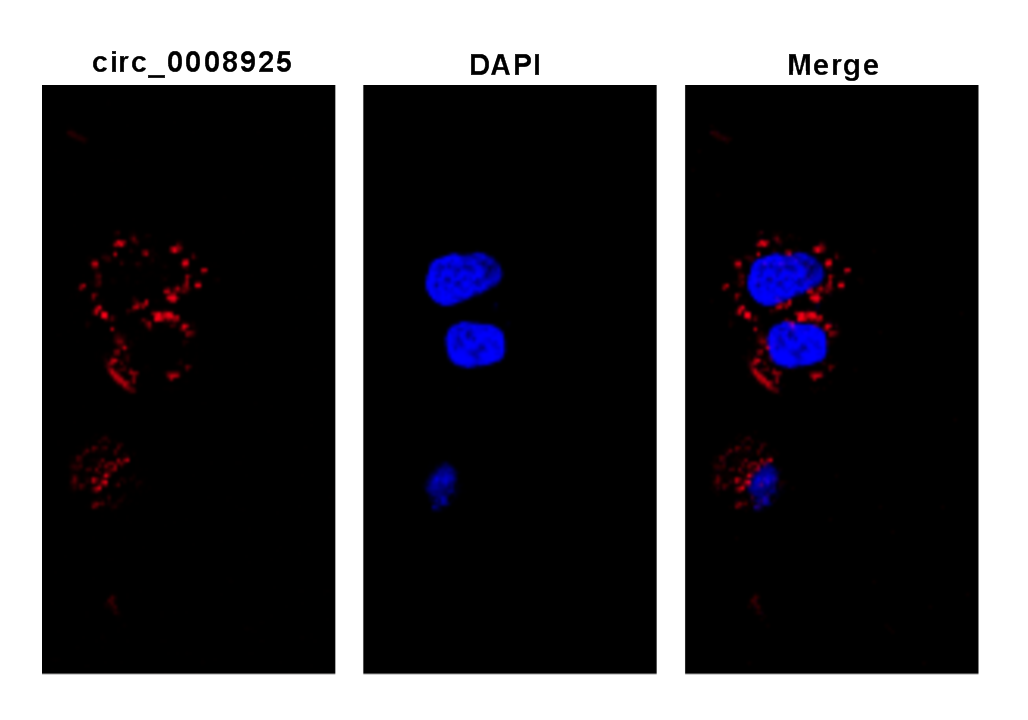

Supplement: Supplementary Figure 1.tif [file IRNF_A_2444393_SM1947.tif]

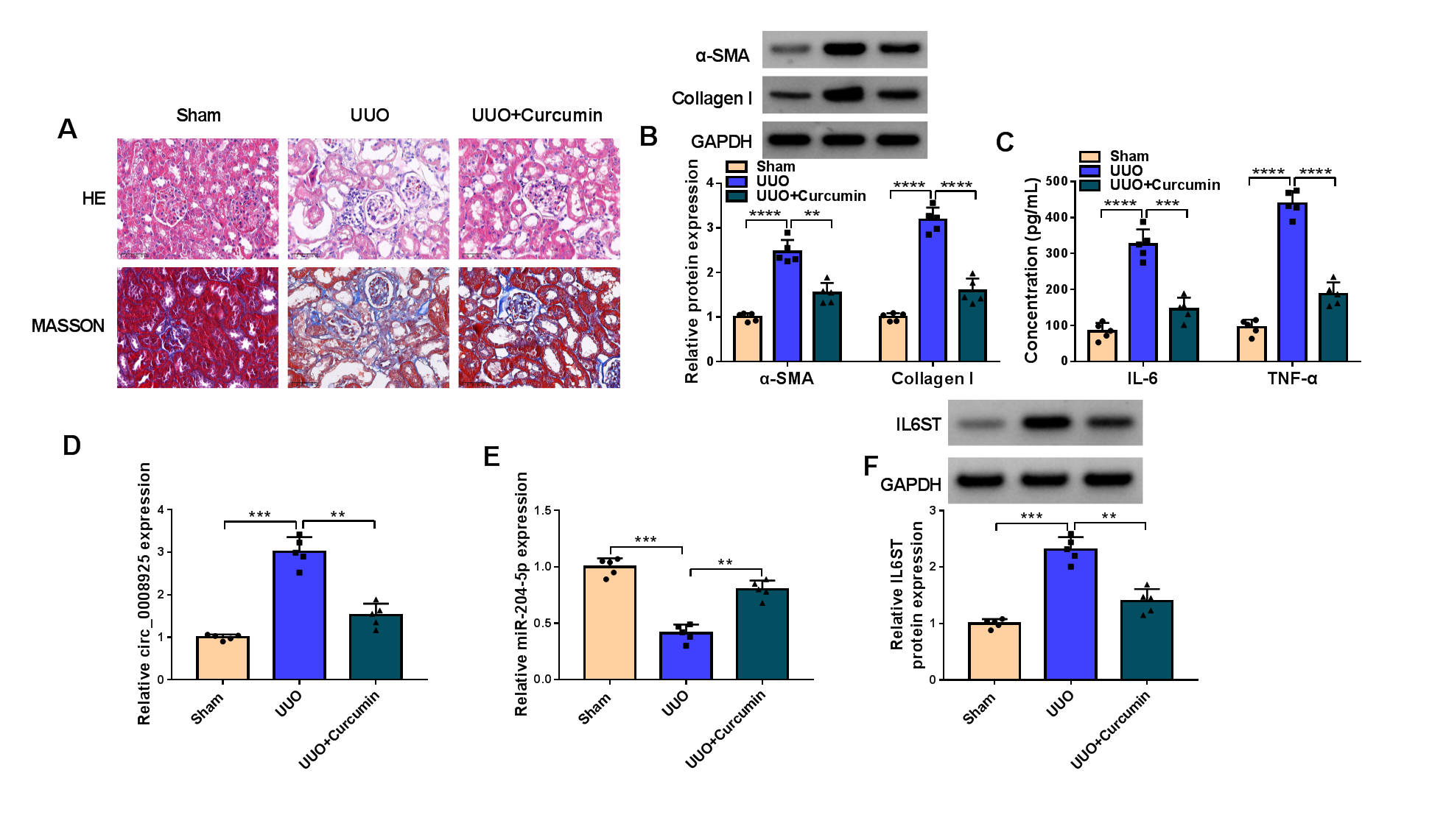

Supplement: Supplementary Figure 2 new.tif [file IRNF_A_2444393_SM1946.tif]

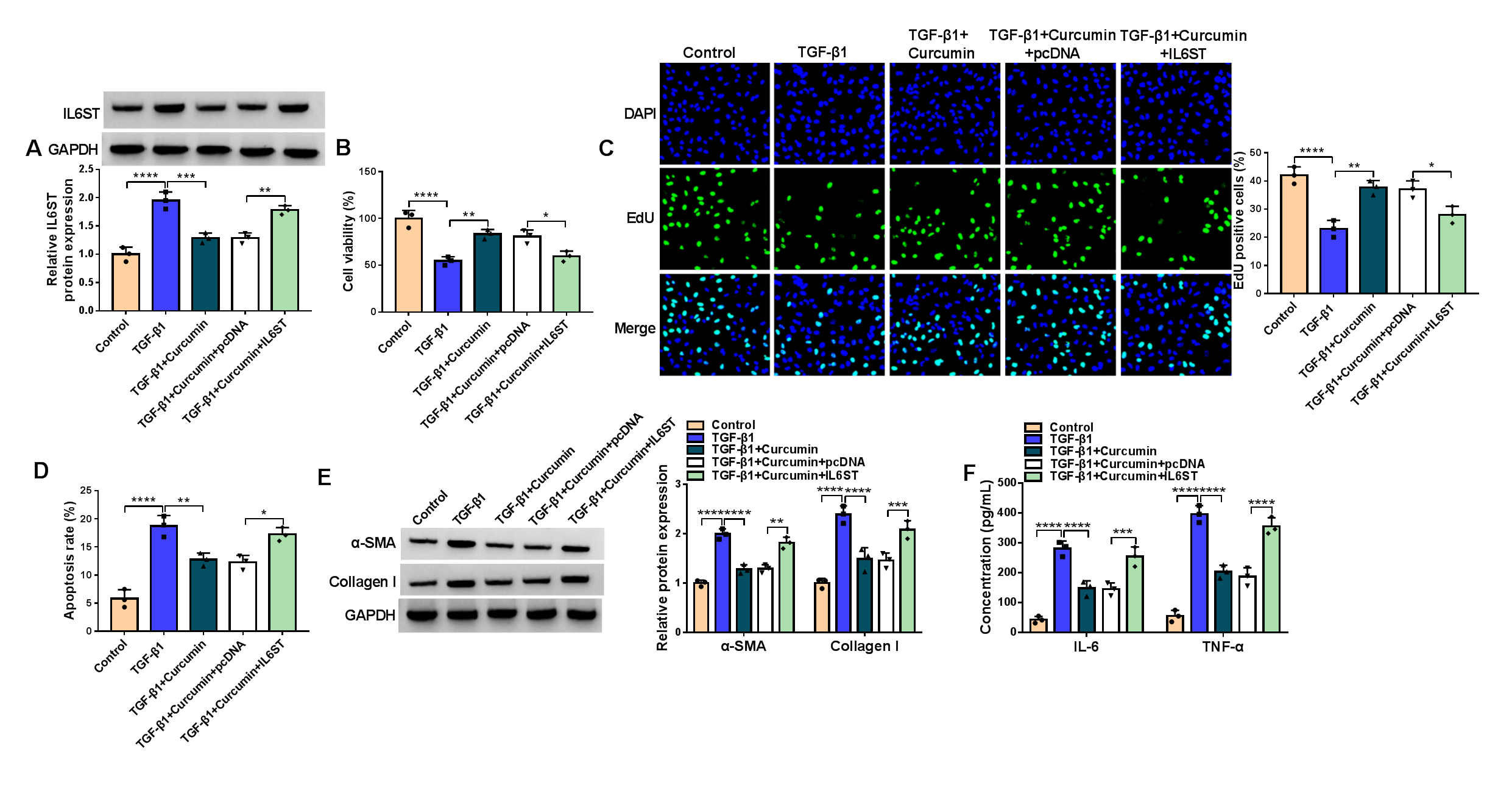

Supplement: Figure 8 revised.tif [file IRNF_A_2444393_SM1945.tif]

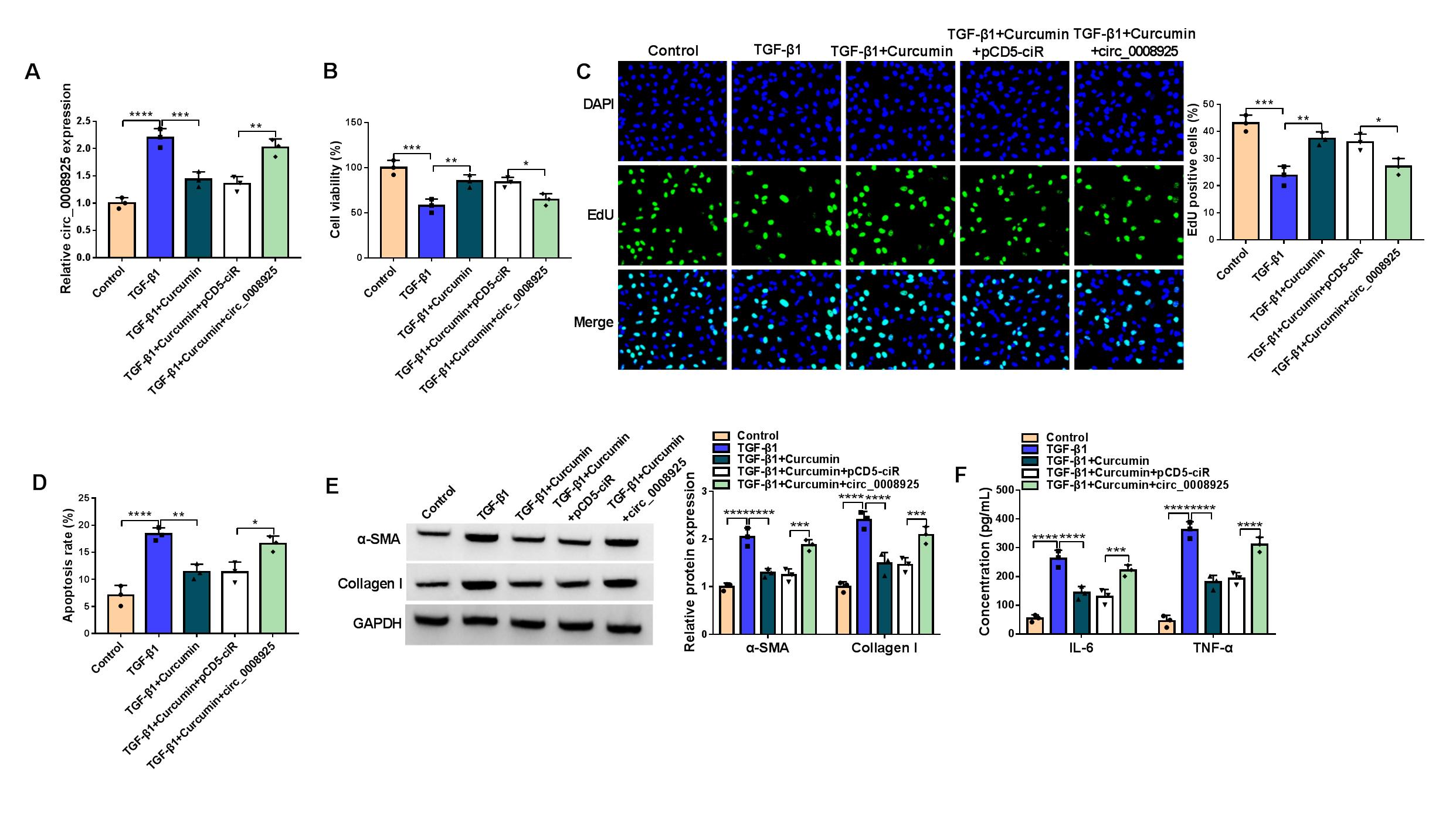

Supplement: Figure 3 revised.tif [file IRNF_A_2444393_SM1944.tif]

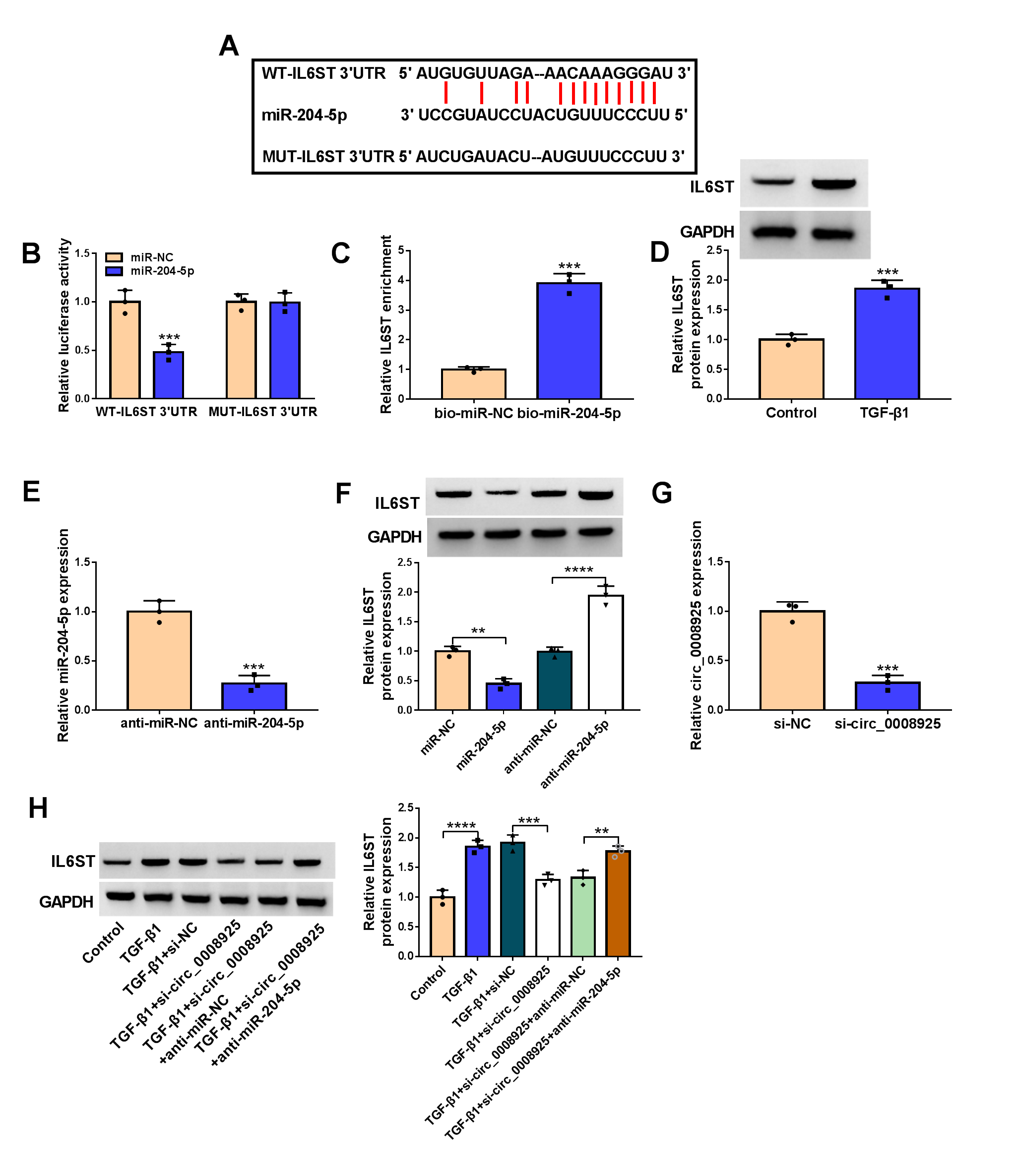

Supplement: Figure 6 revised.tif [file IRNF_A_2444393_SM1943.tif]

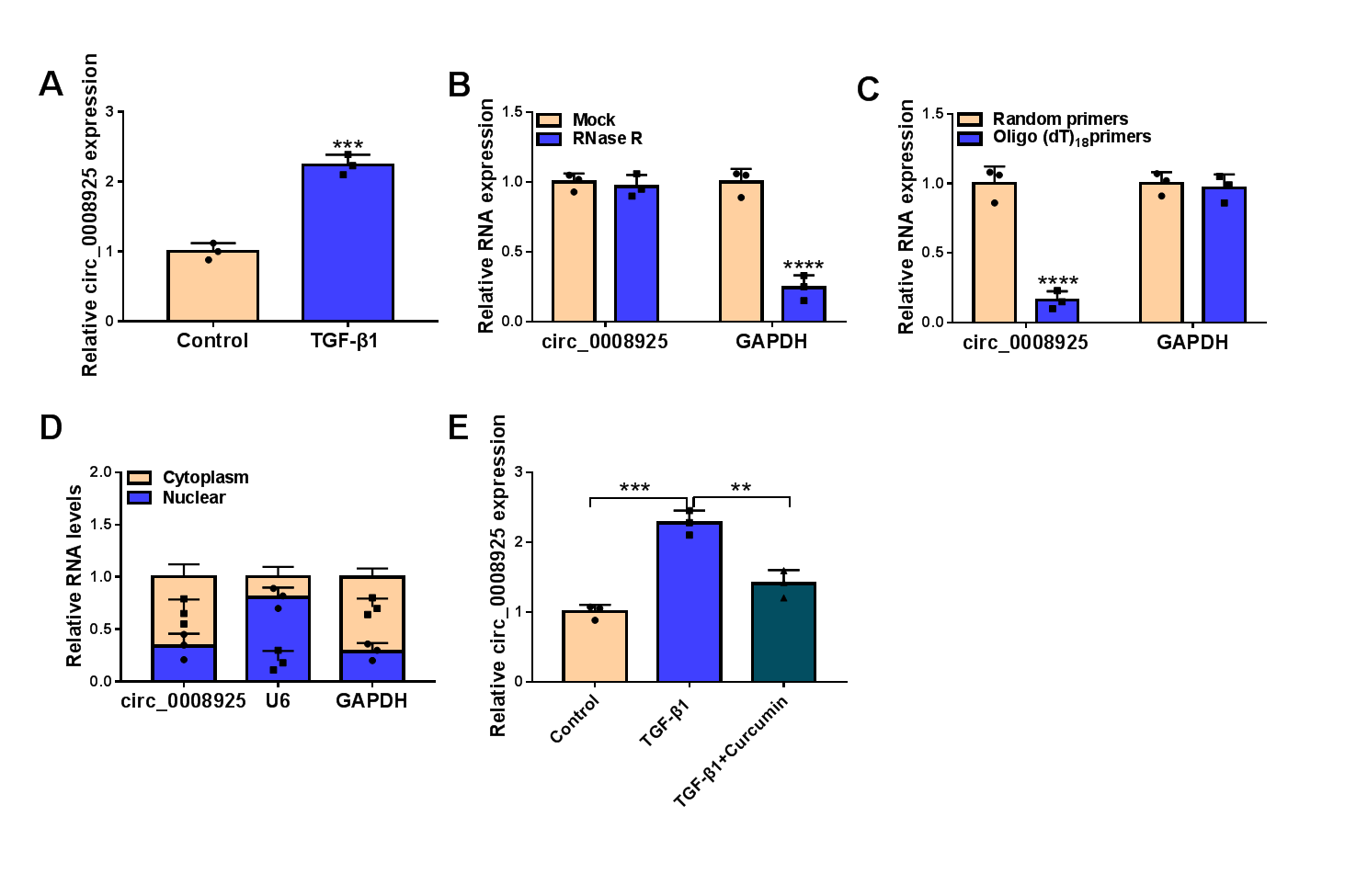

Supplement: Figure 2 revised.tif [file IRNF_A_2444393_SM1942.tif]

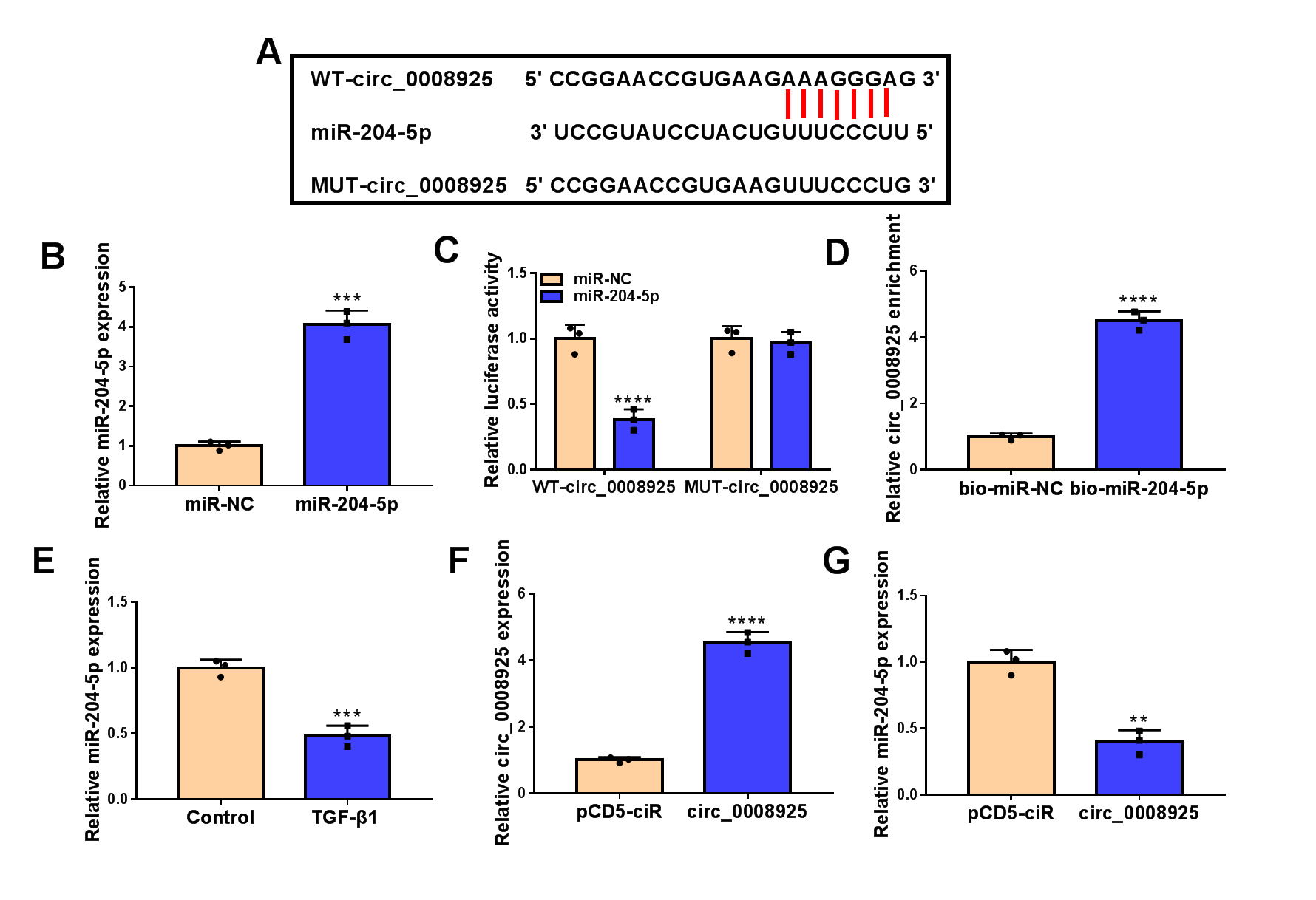

Supplement: Figure 4 revised.tif [file IRNF_A_2444393_SM1941.tif]

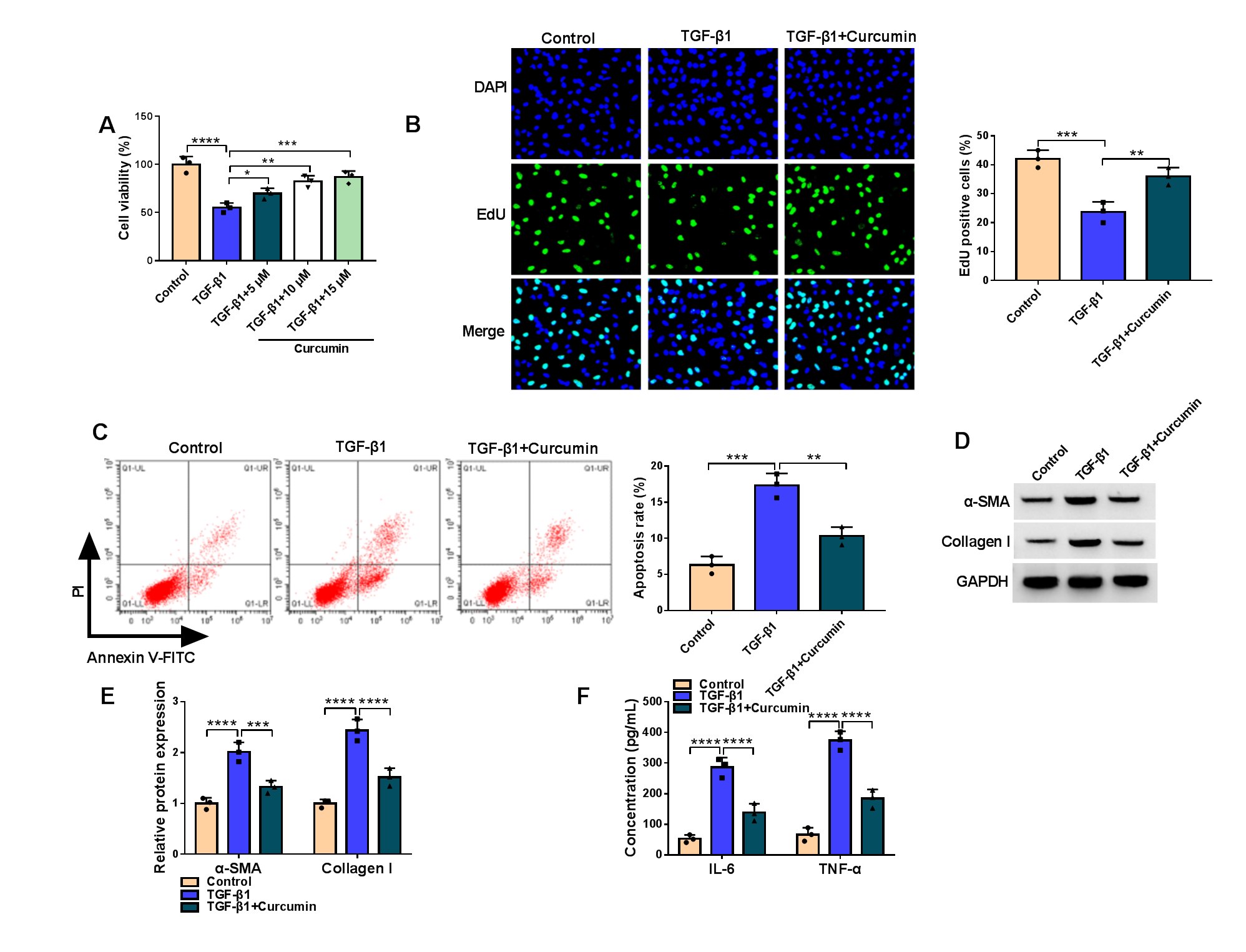

Supplement: Figure 1 revised.tif [file IRNF_A_2444393_SM1940.tif]

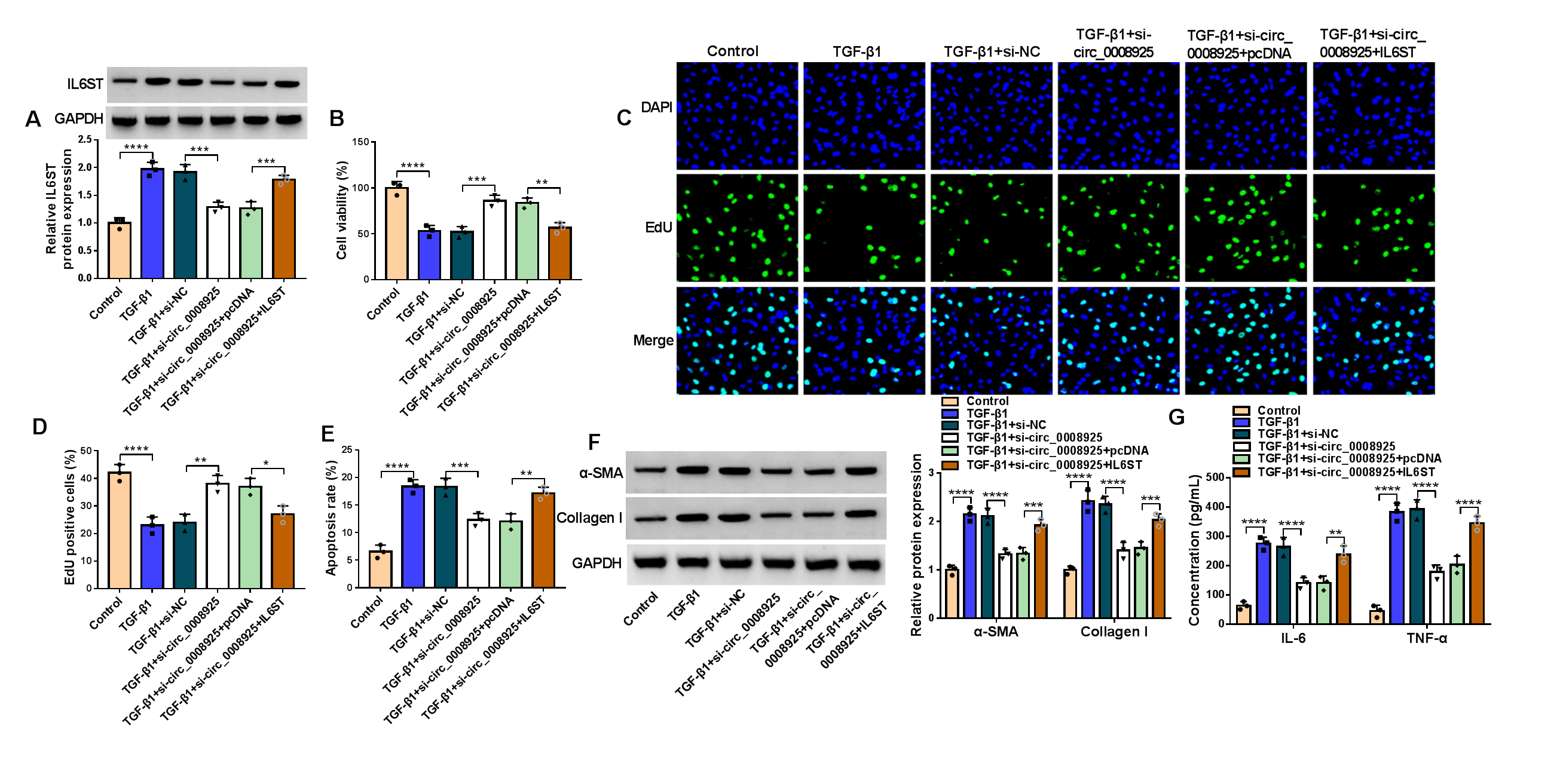

Supplement: Figure 7 revised.tif [file IRNF_A_2444393_SM1939.tif]

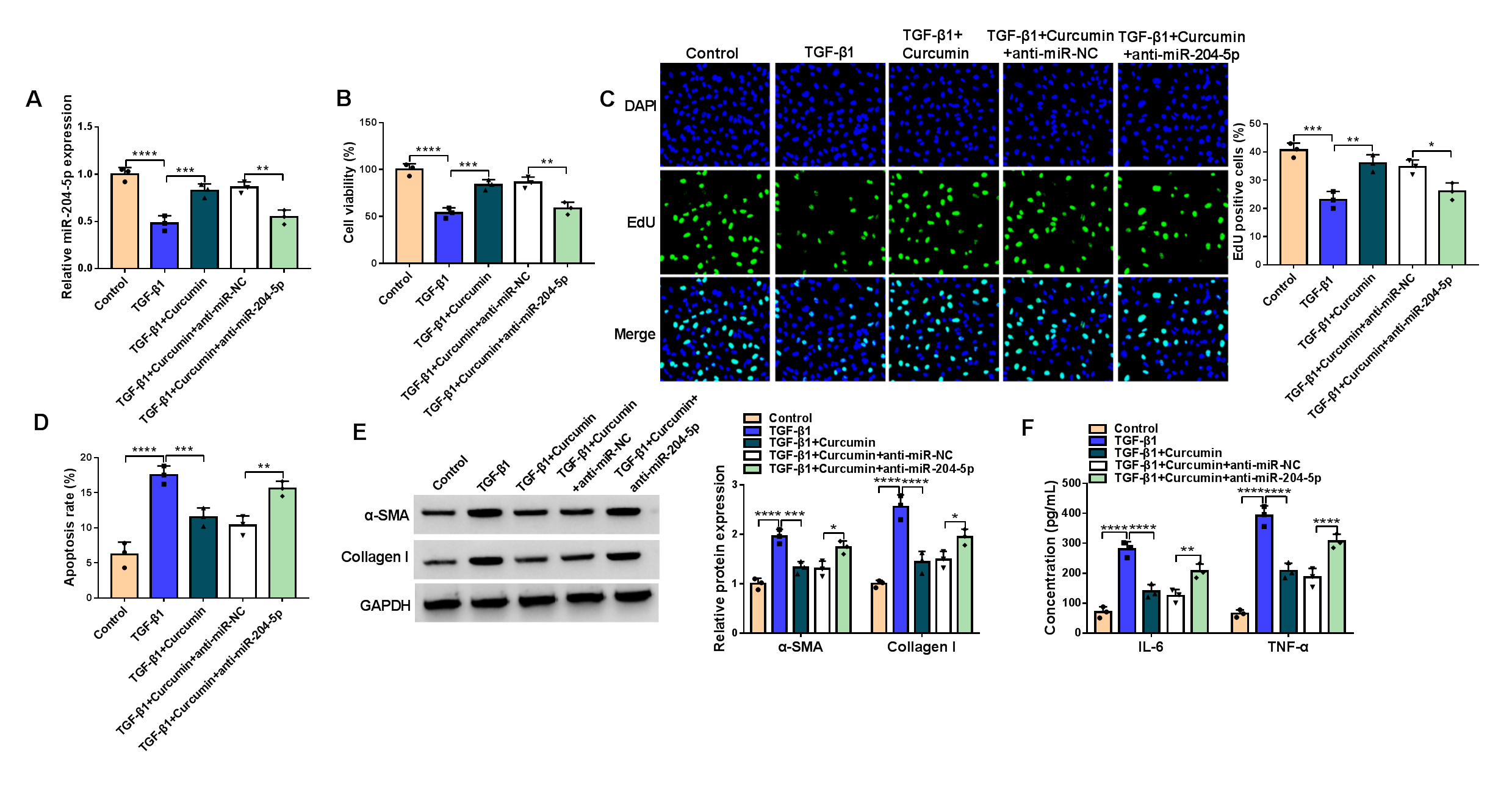

Supplement: Figure 5 revised.tif [file IRNF_A_2444393_SM1938.tif]

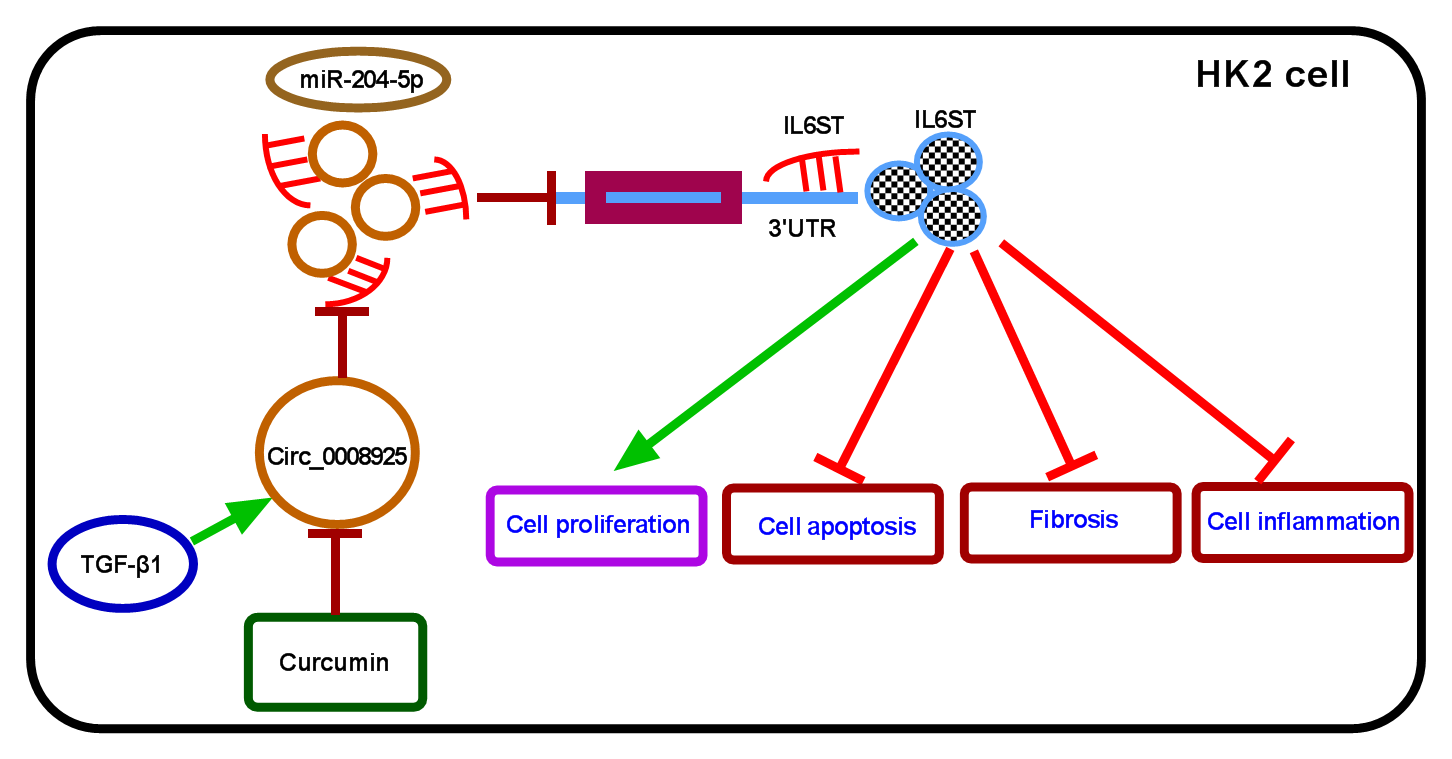

Supplement: Figure 9.tif [file IRNF_A_2444393_SM1937.tif]
