# Supplementary material for: Curcumin alleviates renal fibrosis in chronic kidney disease by targeting the circ_0008925-related pathway
Source: Ren Fail. 2025 Mar 4;47(1):2444393. doi: 10.1080/0886022X.2024.2444393 (PMC11884099; doi:10.1080/0886022X.2024.2444393)
Supplement: All original drawing of WB.pdf [file IRNF_A_2444393_SM1936.pdf]

The original western blots of Fig1

D

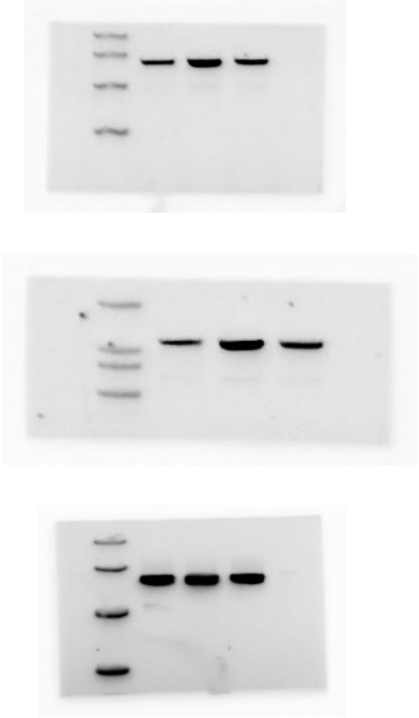

α-SMA

Collagen I

GAPDH

The original western blots of Fig3

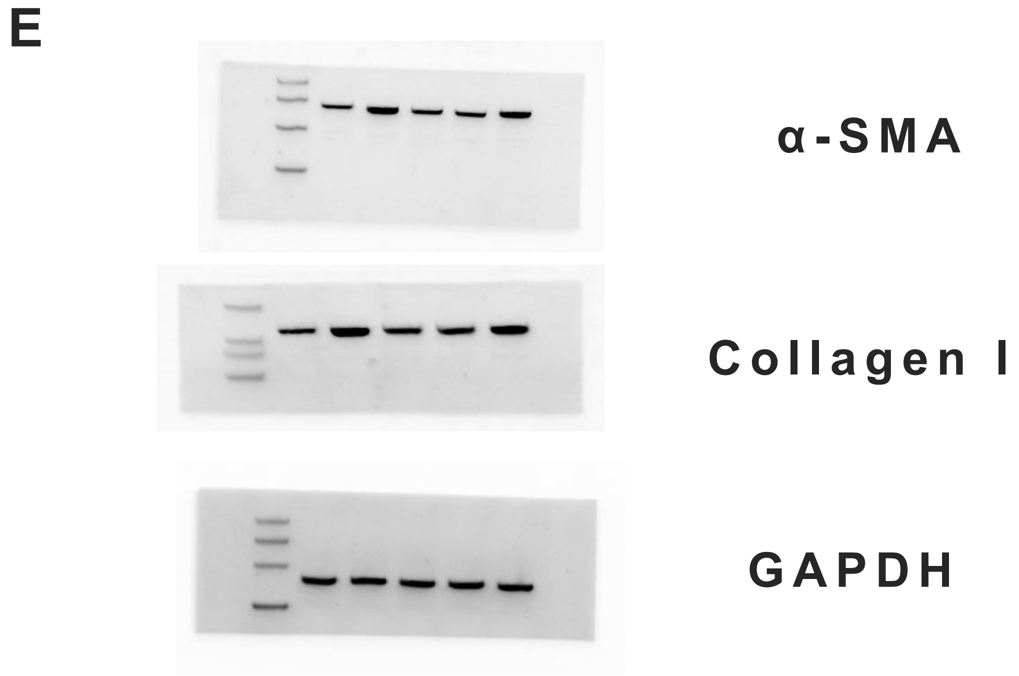

The original western blots of Fig5

E

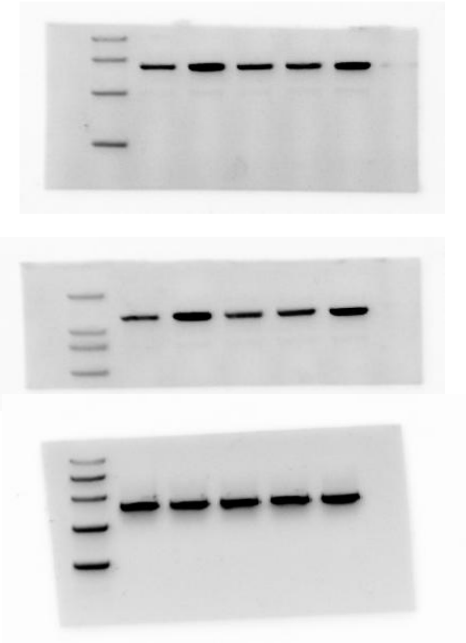

The original western blots of Fig6

D

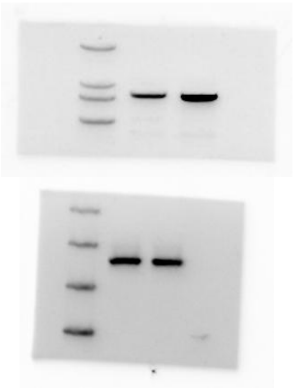

IL6ST

GAPDH

F

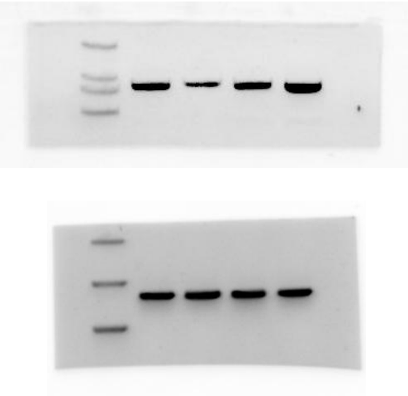

IL6ST

GAPDH

H

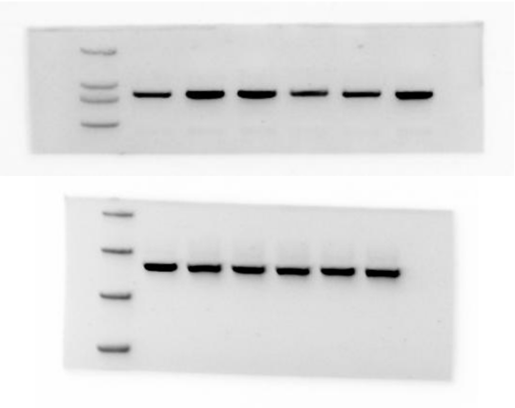

IL6ST

GAPDH

The original western blots of Fig7

A

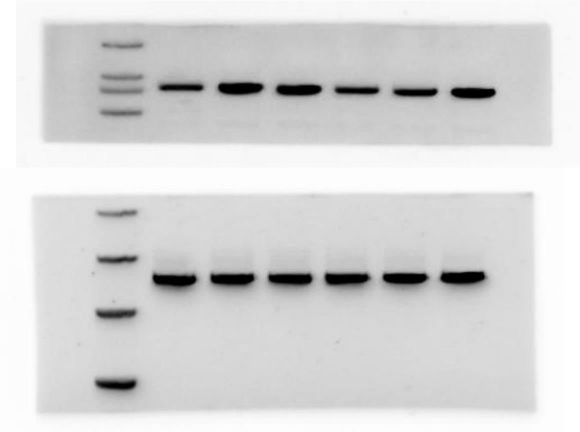

IL6ST

GAPDH

F

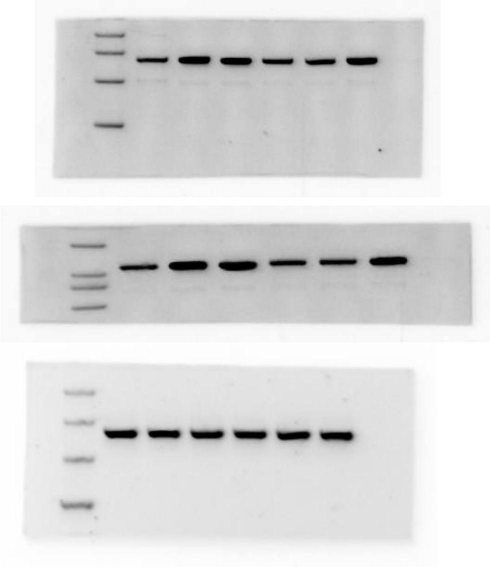

α-SMA

Collagen I

GAPDH

The original western blots of Fig8

A

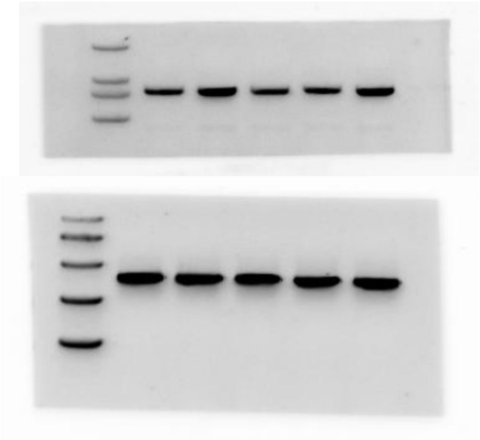

IL6ST

GAPDH

E

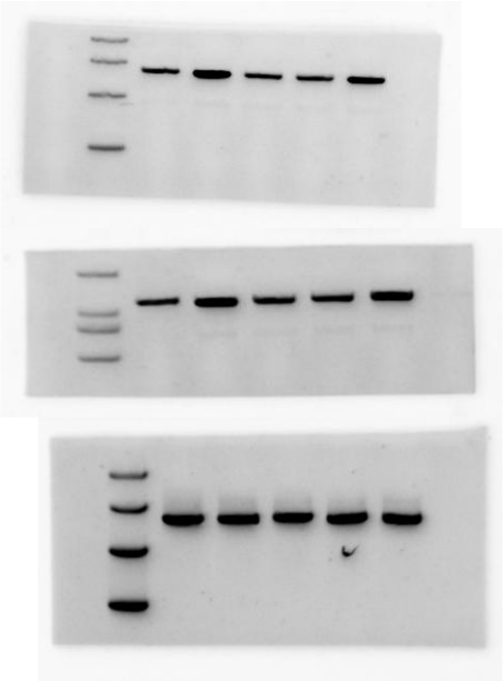

α-SMA

Collagen I

GAPDH

The original western blots of Supplementary Figure 2

B

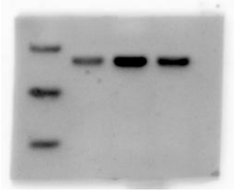

α-SMA

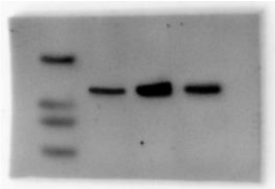

Collagen I

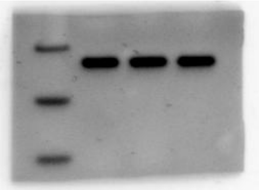

GAPDH

F

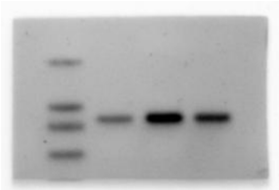

IL6ST

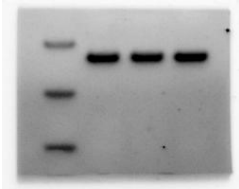

GAPDH
